# Motivations and Constraints to Family Planning: A Qualitative Study in Rwanda’s Southern Kayonza District

**DOI:** 10.9745/GHSP-D-14-00198

**Published:** 2015-05-13

**Authors:** Didi Bertrand Farmer, Leslie Berman, Grace Ryan, Lameck Habumugisha, Paulin Basinga, Cameron Nutt, Francois Kamali, Elias Ngizwenayo, Jacklin St Fleur, Peter Niyigena, Fidele Ngabo, Paul E Farmer, Michael L Rich

**Affiliations:** ^a^​ Partners In Health-Inshuti Mu Buzima, Rwinkwavu, Rwanda; ^b^​ London School of Hygiene and Tropical Medicine, Centre for Global Mental Health, London, UK; ^c^​ National University of Rwanda School of Public Health, Kigali, Rwanda; ^d^​ Partners In Health, Boston, MA, USA; ^e^​ Rwinkwavu District Hospital, Rwinkwavu, Rwanda; ^f^​ Ministry of Health (Rwanda), Kigali, Rwanda; ^g^​ Brigham and Women's Hospital, Division of Global Health Equity, Boston, MA, USA; ^h^​ Harvard Medical School, Department of Global Health and Social Medicine, Boston, MA, USA

## Abstract

**Background:**

While Rwanda has achieved impressive gains in contraceptive coverage, unmet need for family planning is high, and barriers to accessing quality reproductive health services remain. Few studies in Rwanda have qualitatively investigated factors that contribute to family planning use, barriers to care, and quality of services from the community perspective.

**Methods:**

We undertook a qualitative study of community perceptions of reproductive health and family planning in Rwanda’s southern Kayonza district, which has the country’s highest total fertility rate. From October 2011 to December 2012, we conducted interviews with randomly selected male and female community members (n = 96), community health workers (n = 48), and health facility nurses (n = 15), representing all 8 health centers’ catchment areas in the overall catchment area of the district’s Rwinkwavu Hospital. We then carried out a directed content analysis to identify key themes and triangulate findings across methods and informant groups.

**Results:**

Key themes emerged across interviews surrounding: (1) fertility beliefs: participants recognized the benefits of family planning but often desired larger families for cultural and historical reasons; (2) social pressures and gender roles: young and unmarried women faced significant stigma and husbands exerted decision-making power, but many husbands did not have a good understanding of family planning because they perceived it as a woman’s matter; (3) barriers to accessing high-quality services: out-of-pocket costs, stock-outs, limited method choice, and long waiting times but short consultations at facilities were common complaints; (4) side effects: poor management and rumors and fears of side effects affected contraceptive use. These themes recurred throughout many participant narratives and influenced reproductive health decision making, including enrollment and retention in family planning programs.

**Conclusions:**

As Rwanda continues to refine its family planning policies and programs, it will be critical to address community perceptions around fertility and desired family size, health worker shortages, and stock-outs, as well as to engage men and boys, improve training and mentorship of health workers to provide quality services, and clarify and enforce national policies about payment for services at the local level.

## Abstract (precis)

Community members and health workers recognized the value of spacing and limiting births but a variety of traditional and gender norms constrain their use of contraception. Limited method choice, persistent side effects, transportation fees, stock-outs, long wait times, and hidden service costs also inhibit contraceptive use.

## INTRODUCTION

At the 2012 London Summit on Family Planning, donors and national governments committed to providing access to modern contraceptives by 2020 to an additional 120 million women around the world who have unmet need for family planning, as well as to focusing on the human rights of women and girls and to launching a reinvigorated global platform for achieving universal access to family planning. Yet this renewed commitment also highlighted the challenges of achieving universal access to family planning, including in countries such as Rwanda that have seen impressive gains.

Rwanda is among the most densely populated countries in Africa; between 2002 and 2012, the average annual population growth was 2.6%.^1^ Population pressures are compounded by the country’s young population, with 61.5% under the age 25 as of 2010,^1^ highlighting the importance of ensuring access to sexual and reproductive health (SRH) services as the majority of the population enters its reproductive years.

Rwanda’s ambitious national development plan, *Vision 2020*, outlines a framework to improve population welfare, including a goal of increasing contraceptive coverage among married women to 70% by 2015.^2^ Rwanda’s *National Reproductive Health Policy*^3^ and *National Family Planning Policy*^4^^,^^5^ provide a platform for expanding access to modern contraceptives and strengthening service delivery. Further, mass media information campaigns, alongside the decentralization of family planning services to health centers and by Rwanda’s roughly 45,000 community health workers (CHWs),^6^ have brought behavior change messages close to home, promoting reproductive choice, birth timing, and birth spacing.

**FIGURE 1.**
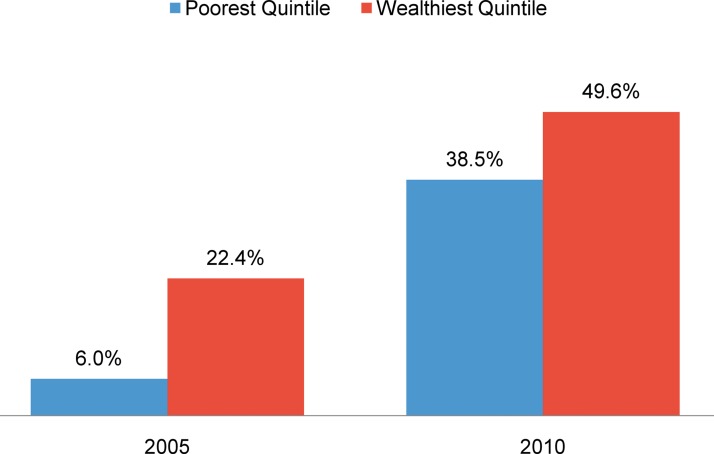
Use of Modern Contraceptives Among Married Women Ages 15–49 in Rwanda, by Wealth Quintile, 2005 and 2010 Source: Rwanda Demographic and Health Surveys.^7^

Rwanda reports significant gains in contraceptive coverage since 2000. According to the Rwanda Demographic and Health Survey (DHS), use of modern contraception among married women has risen from 5.7% in 2000 to 45.1% in 2010.^7^ This increase in contraceptive prevalence has been accompanied by a decrease in the estimated total fertility rate (TFR), from 6.1 in 2005 to 4.6 in 2010,^7^ attributed in part by the World Bank to improved education and housing for women.^8^ Rwanda’s 2012 census showed a further decline in the TFR to 4.0 that year and an average annual rate of decline in the TFR of 6.0% since 2005.^1^ Equity gaps in contraceptive use between the wealthiest and poorest women also appear to have shrunk considerably (Figure 1). Furthermore, reported family size preferences among women fell from an average of 4.9 children in 2000 to 3.3 in 2010.^7^ Future gains thus seem likely, if access to and retention in family planning services are adequately supported. Yet despite these gains, there remains high unmet need for contraception, at 19% of married women,^7^ and households continue to face barriers to accessing quality family planning services, including physical access, cost, lack of accurate information, limited knowledge, side effects, and partner communication.^9^^–^^13^

Modern contraceptive use among married women in Rwanda rose from about 6% in 2000 to 45% in 2010.

In order to increase uptake and promote retention in family planning programs, it is critical to understand local perspectives on birth, fertility, contraception, and family composition. While social norms have been shown to affect both contraceptive demand and family planning practice in Rwanda,^8^ relatively few studies have undertaken qualitative research to investigate factors that affect family planning use in communities, with some notable exceptions.^12^ Several recently published studies have provided important qualitative insight into barriers to accessing contraception and benefits of long-acting methods.^13^^,^^14^ This study adds further exploration of the underlying factors contributing to women’s and men’s perceptions of family planning within broader understandings of SRH.

The Department of Community Health and Social Development of the NGO Partners In Health-Inshuti Mu Buzima (PIH-IMB) undertook a broad qualitative investigation to explore general public perceptions of reproductive health and family planning in southern Kayonza district, a rural area in the country’s eastern province. The study was conducted in partnership with Rwinkwavu District Hospital and the Maternal and Child Health Unit of the Ministry of Health. The main foci of the research included: (1) community perceptions of reproductive health and family planning; (2) quality of services; (3) adherence to and discontinuation of contraception; and (4) barriers to accessing family planning and reproductive health services. This paper describes findings from the first phase of research, which was comprised of interviews with health care providers, CHWs, and community members.

## METHODS

### Population and Setting

Since 2005, PIH-IMB has worked in partnership with the Rwandan Ministry of Health to provide an enhanced package of health services and support in 3 rural districts in Rwanda. In southern Kayonza district, PIH-IMB works in collaboration with Rwinkwavu District Hospital, 8 health centers, and approximately 1,000 CHWs.

**FIGURE 2.**
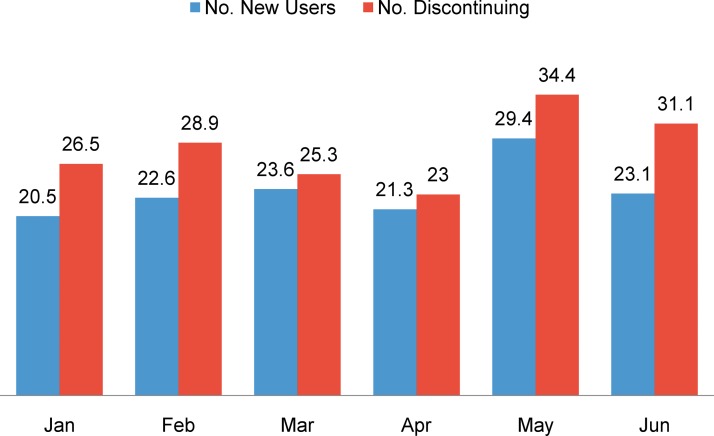
Average Number of New Contraceptive Users and Discontinuers Across All 8 Health Centers in Southern Kayonza District, January–June 2012 Source: Rwanda Health Management Information System.^15^

As of 2012, southern Kayonza had a population of 188,363 individuals, including 44,681 women of reproductive age (15–49 years old).^1^ In addition to national media campaigns, information on SRH is available at the community level through village meetings and home-based counseling by CHWs. Family planning services are available at each of the 8 health centers in the catchment area, as well as at the community level through CHWs as part of the national decentralization strategy. Rwanda’s southern Kayonza district has a high rate of enrollment in the family planning program, similar to other parts of Rwanda,^7^ but also has high rates of contraceptive discontinuation (Figure 2). Given the roughly equal proportion of facility clients who continue and discontinue contraception each month, the critical importance of understanding why women discontinue use was taken into consideration in our study design.

The eastern province in which southern Kayonza is located has Rwanda’s highest total fertility rate and adolescent fertility rate, with 16% of women giving birth by the age of 19.^15^ Fertility in the eastern province has also fallen more slowly in recent years than in other provinces, a trend that the National Institute of Statistics of Rwanda attributes in part to the relatively young population of the region.^16^

The eastern province in which southern Kayonza is located has Rwanda’s highest total fertility rate.

### Recruitment and Data Collection

Between October 2011 and December 2012, a PIH-IMB research team conducted a series of semi-structured interviews with adult participants. Interview discussion guides included questions on knowledge of and beliefs regarding fertility, reproductive health, and family planning; the role of religious and other local leaders and health care providers in promoting family planning; experiences using contraceptive methods; and interpersonal relationships with friends, spouses, relatives, and health care providers.

Adult participants were recruited from all 8 health centers’ catchment areas in southern Kayonza district. The family planning nurse and maternity nurse were interviewed at each health center. Three villages per health center catchment area were then selected at random, and the 2 maternal and child health CHWs in each village were interviewed. These CHWs keep logs of all households in their catchment areas, including the name and age of each household member. The names of all adults aged 18 or over were compiled from these household logs into a single list, from which 1 male and 1 female adult community member per village were randomly selected for interview. In order to explore the diverse perspectives of community members in southern Kayonza, no upper age limit was imposed.

Participants were compensated 2,000 Rwandan Francs (approximately US$3) for their time. All participants provided informed consent. All focus groups and interviews were audio-recorded and conducted in the local language, Kinyarwanda, by a team of trained data officers.

### Ethical Approval

This study received approval from the Rwanda National Ethics Committee in Kigali, Rwanda, and from the Brigham and Women’s Hospital Partners Human Research Committee in Boston, Massachusetts, USA.

### Data Analysis

Data were transcribed in Kinyarwanda and translated into English. Data were analyzed using NVivo and Dedoose software. The PIH-IMB research team read through and *in vivo* coded all transcripts to develop lists of preliminary themes using participants’ own words. The research team met regularly to discuss emerging themes and to develop a formal codebook, which linked each content area with specific thematic codes. The team then recoded the transcripts following the content areas outlined in the codebook. Seven themes emerged as the key analytical categories under which the team grouped related subcodes. Regular meetings among the coding team allowed discussion of any discrepancies in code definitions and in the application of codes, ensuring the reliability of coded data and consistent coding of transcripts. Identifying information was omitted during data analysis to protect participant confidentiality.

## RESULTS

### Characteristics of Study Participants

In total, we recruited 159 participants: 15 family planning and maternity nurses based at health centers, 48 maternal and child health CHWs, and 96 community members. The mean age of the 96 community members was 38 years (Table), with a range of 20 to 87 years. Community members reported an average of 4 children, and 86.5% were either married or cohabitating. Most (86.5%) were subsistence farmers, and they had, on average, 4 years of schooling (over half had not completed primary school).

**TABLE.** Demographic Characteristics of Study Participants, Kayonza District, Rwanda (N = 159)

	**Community Members (n = 96)**	**Community Health Workers (n = 48)**	**Nurses (n = 15)**
Age, mean, y	38	38	31
Sex, No. (%)			
Male	48 (50.0)	24 (50.0)	7 (46.7)
Female	48 (50.0)	24 (50.0)	8 (53.3)
No. of children, mean	4	4	1.5
No. of years of school, mean	4	7	12
Marital status, No. (%)			
Single/unmarried	4 (4.2)	6 (12.5)	4 (26.6)
Married/cohabitating	83 (86.5)	42 (87.5)	11 (73.3)
Other	9 (9.3)	0 (0.0)	0 (0.0)
Occupation of community members,^a^ No. (%)			
Farmer	83 (86.5)	--	--
Other	13 (13.5)	--	--

^a^​ Community health workers were not asked to describe employment in addition to community health work.

**FIGURE 3.**
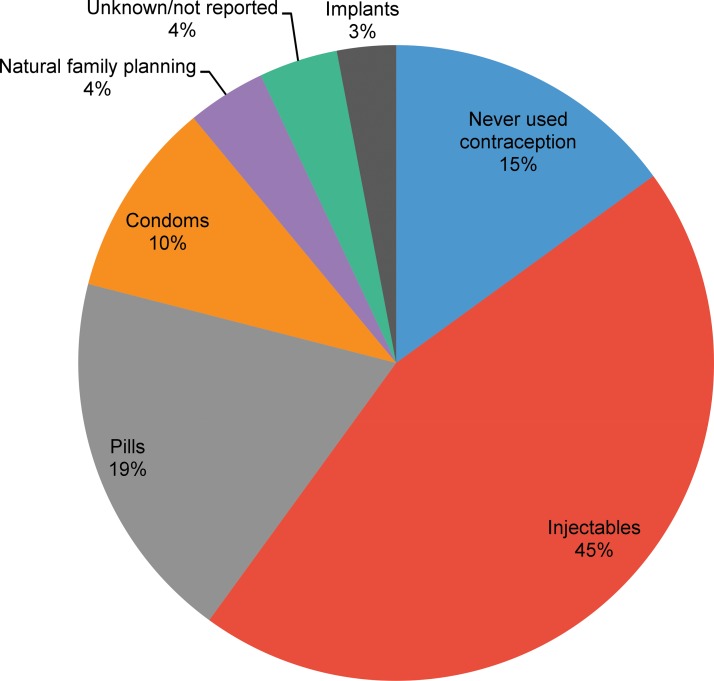
Current Contraceptive Use Among All Participants^a^ (N=159) ^a^ Current contraceptive method use was self-reported at the time of the interview either by female participants or by male participants about their partner or spouse.

Of the 159 adult participants, 15% (15 community members, 6 CHWs, and 3 health facility nurses) reported never using contraception (Figure 3). Among those who said they (or their partners, in the case of male participants) had used modern contraceptive methods, the most commonly reported contraceptive method currently used was injectables at 45%, followed by pills (19%) and condoms (10%). Implants were used by 3% of the participants and the other long-acting and permanent methods were not used at all.

### Four Main Themes Influencing Reproductive Health Decision Making

**FIGURE 4.**
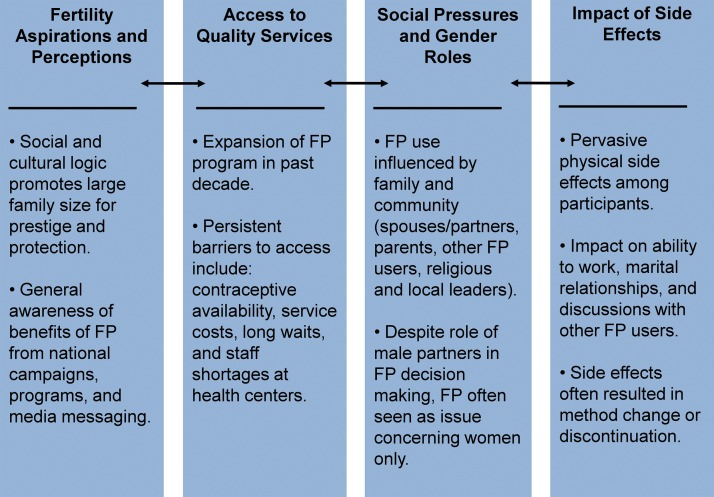
Key Family Planning (FP) Themes Emerging From Interviews

Four key themes emerged from the interviews: (1) fertility aspirations and perceptions of family planning, (2) social pressures and gender roles, (3) access to quality services, and (4) impact of side effects (Figure 4). These themes were interwoven throughout participants’ narratives, influencing reproductive health decision making, including enrollment and retention in family planning programs.

Participants exhibited general awareness of family planning and its benefits; however, many also shared ambitions for large families and related experiences cycling in and out of the family planning program as they navigated between these fertility aspirations. While family planning services are widely available at health centers, participants continued to face barriers to accessing high-quality services, and persistent side effects also affected family planning use. Gender roles and social networks further exerted pressure on family planning use. When spouses, friends, or parents did not support family planning use, many women considered enrolling in the program secretly and without familial support.

#### Fertility Aspirations and Perceptions of Family Planning

All participants had some knowledge of contraceptive methods, and many had learned about family planning through national messaging, echoing findings from the 2010 Rwanda DHS, in which 70% of married women had heard a family planning message on the radio or television or in the newspaper.^17^ Participants often viewed family planning as a means of improving the socioeconomic condition of one’s family and community, explaining that contraception facilitates birth spacing and timing, which can, in turn, improve marital relations, sanitation, and hygiene; give a mother time to work and care for her children; and lead to better health outcomes for pregnant women. Further, participants shared that using contraception allows a family to save money in order to feed and clothe their children and to pay school and health insurance fees:

*Family planning is good … [without family planning] you can give birth to many children and your life becomes very complicated. Now I have only 2 children and their life is very different from if I’d had 5 children. When you give birth to many children … they need soap and clothing and they will often be dirty. So for me, family planning use was good and I would even educate those who are not yet using family planning to start.* (Female community member, 38 years old)

Many participants had learned about family planning through national messaging.

Community members explained that their health care providers and CHWs counseled them about a range of methods. Participants explained that injectables (the most commonly used method among study participants) are preferable to other methods because it is easier to get an injection every 3 months than to take a pill every day. As 1 participant explained:

*You know taking pills is not easy, since you are always working in your fields, you may forget to swallow your pills … so that is the reason why my wife decided to choose to use injections.* (Male CHW, 36 years old)

Participants preferred injectables because they thought it was easier to get an injection every 3 months than to take a pill every day.

Often condoms were used after an unsuccessful trial of hormonal methods. Natural methods such as withdrawal, CycleBeads, and the traditional calendar method were common among participants whose religious beliefs prohibited them from using modern contraception. While some participants had heard of long-acting methods, such as intrauterine devices and vasectomy, these methods were used by few participants and were not routinely offered at all health centers in southern Kayonza district.

Long-acting and permanent methods were not routinely offered at all health centers in the district.

Family planning was also seen by many as a mechanism for contributing to the nation’s development by limiting population strain and land shortages, thereby reducing poverty:

*When we are in community meetings, they tell us about family planning and about the problems of overpopulation in our country.* (Male community member, 47 years old)

Another participant explained:

*I don’t believe that there are disadvantages to using family planning because we live in different times today than we did in the past. People used to have large plots of land in the past and parents had land to give to their children. But today people have no land to give to their children.* (Male community member, 48 years old)

In addition to reflecting on land shortages and population changes, participants also reflected on changes in the availability and promotion of family planning information and services over time. The 1994 Rwandan genocide often figured prominently in these reflections, as a key turning point in the country’s history that reshaped the political and social landscape:

*In our time there, family planning was not known everywhere. People hear about it now after the genocide. Before the genocide no one used family planning.* (Female community member, 58 years old)

While these changes were often recounted first-hand by older participants, a narrative of change over time in population, land availability, and increased family planning access was also echoed by younger participants.

Although participants readily discussed the benefits of family planning, many also valued large families and high parity. In addition to serving as a status symbol, participants viewed large families as a form of insurance in a context where many had witnessed the loss of a child from childhood illness, due to birth complications, or during the 1994 genocide. As many participants in our study recounted experiences of losing children, having multiple children was often discussed as a means of ensuring the longevity of one’s family in the face of future uncertainty. One participant shared:

*Rwandans say that if you give birth to few children, what will happen if they die?* (Female community member, 58 years old)

Yet she continued:

But don’t think like that … Today there is malnourishment everywhere. We don’t have any land left to cultivate and farm. Many don’t eat all day and all night, and children often become homeless … For this reason, family planning is good … You should give birth to few children that you are able to bring up properly.

Although participants recognized the benefits of family planning, many also valued large families.

Through such narratives, participants weighed the benefits of family planning alongside desires for large families and recounted experiences cycling in and out of the family planning program as they reconciled these vying fertility aspirations.

#### Social Pressures and Gender Roles

Gender roles and relationships with family members were critical factors in women’s decisions to enroll in the family planning program and structured beliefs about childbirth and fertility. Many female participants explained that without a husband’s approval, contraception had to be used in secret. At the same time, family planning was also described as a woman’s matter with which men should not be concerned:

*The problem is that men feel that family planning is only for women … No men ever come to the health center asking for family planning. Men say that family planning is just for women.* (Female CHW, 48 years old)

Participants described family planning as a woman’s matter, but men often exerted power when making reproductive decisions.

As a result, CHWs and health facility staff explained that a large part of their role in promoting family planning was educating men on the importance of family planning:

*Sometimes, husbands and wives disagree. We try to teach men so that they will see that family planning is necessary. But some men don’t understand. They say that they don’t enjoy sexual intercourse when using contraception.* (Female CHW, 50 years old)

CHWs employed many techniques for reaching male community members, for example, by interacting with men during household visits and at community meetings. In interviews, male CHWs shared that part of their role was acting as an ambassador for promoting family planning to other men in the community, often teaching by example, starting with their own households:

*I educated my wife about family planning and she understood it.* (Male CHW, 47 years old)

Gendered behavior further influenced reproductive health decision making, as unmarried women were often discouraged from accessing contraceptive services at health centers, both within their communities and by providers themselves. For example, informal rules enforced at some health centers required women to be accompanied by male partners. Participants described both marital status and age as important factors affecting access to contraception. Unmarried and young women often faced stigma if they were interested in accessing family planning services and would rarely go to the health center for fear of judgment—specifically, fear of being labeled “prostitutes.” Some providers shared the stigmatizing attitudes of community members:

*People who aren’t married yet don’t use family planning, except for those who are prostitutes. Prostitutes are the only ones who come to get family planning.* (Female CHW, 42 years old)

Young and unmarried women often faced stigma when accessing family planning services.

CHWs reported that on rare occasions young people would seek them out to ask questions and access contraception, although this behavior was more common among young men than women:

*A few young men come to me to ask me for condoms, but as for young women, I have to go looking for them because they think that if people know that they use family planning, people will think they are prostitutes. … Recently, only one young man came to ask me for condoms. I gave them to him, but no young woman has ever come to see me. Maybe they [young women] go to the health center, but generally they are too embarrassed. They explain that they can’t go to get family planning when they aren’t married.* (Female CHW, 50 years old)

While social pressure in some cases acted as a barrier to access, local leaders and CHWs also used social strategies to reinforce messages surrounding the benefits of family planning use. In community meetings at the village level, local leaders advocated family planning use, and CHWs made home visits and held meetings encouraging families to enroll in the program. Religious institutions offered mixed messages. While some religious leaders endorsed family planning, others promoted only natural methods, and still others prohibited all family planning use:

*Religious leaders do talk about family planning. And some Christians do use family planning methods, but there are others who don’t use family planning. Not all people place the same value on family planning.* (Male community member, 45 years old)

Some religious leaders endorsed family planning while others did not or promoted only natural methods.

#### Access to Quality Services

Despite national gains in contraceptive availability over the past decades,^17^ participants recounted many barriers remaining that inhibited widespread access to quality family planning services, such as transportation, variable quality of care, lack of diversity in the contraceptive methods available at health centers, and costs associated with services. Lack of transport and long wait times upon arrival at the health center, combined with the opportunity cost of lost work, often made going to a health center prohibitive for women in southern Kayonza. Stock-outs at health centers also impacted the quality of services women received:

*The biggest challenge is stock-outs of medicines and related materials … And many women stop using contraception, not because they’ve decided to but because of stock-outs. You must understand that when stock-outs occur, women can become pregnant while waiting for contraceptives to be delivered to the health center.* (Female nurse, 28 years old)

Many barriers inhibited widespread access to family planning, including stock-outs.

While family planning services at health centers are free of change, some participants shared that if they did not have *mutuelle de santé* (“mutual health,” Rwanda’s community-based health insurance system),^18^ they felt they could not go to the health center to receive services. Further, despite policies meant to eliminate the cost of family planning services, community members were often asked to make co-payments if they returned to the health center to change methods or to receive monthly contraceptive services:

*When people come for a family planning method, they are asked to pass through the mutuelle de santé office instead of coming directly to the family planning office. And when they go to the mutuelle office, they are asked to pay 200 Rwandan Francs [about US$0.29], when it is known that family planning services are free of charge.* (Male nurse, 41 years old)

#### Impact of Side Effects

Side effects were a salient feature in nearly all interviews with community members, influencing women’s decisions to adhere to or discontinue contraceptive use and impacting interpersonal relationships, work productivity, and marital relationships. In the context of minimal counseling, long waits at health centers, and fees for services, many women discontinued their contraceptive methods when side effects persisted, rather than returning to the health center for additional consultations. Participants experienced a range of side effects that they associated with hormonal contraceptive methods, including excessive bleeding, weight fluctuation, headaches and backaches, low libido, and vaginal dryness:

*Many women deal with frequent side effects. When we go to see them, some tell us that they have a headache or a backache. Another side effect women experience is bleeding heavily; they won’t stop bleeding for an entire month. Others say that they no longer menstruate.* (Female CHW, 42 years old)

When women experienced persistent side effects, many discontinued their method rather than return to the health clinic.

These side effects affected women’s ability to work and their marital relationships. As 1 participant shared:

*Family planning was killing me. I suffered from headaches, and I would spend like 2 days non-stop with a headache. I suffered from backaches and I could not do hard work.* (Female community member, 39 years old)

Side effects also impacted marital relationships when women felt concerned they were no longer able to please their husbands or maintain their regular sexual activity:

*Some women who use family planning begin to have headaches. Later, they may experience low sex drive or may not seem sexually desirable to their husbands. And this can cause misunderstandings between husbands and wives … Women may choose to discontinue using family planning after struggling every day with their husbands. When it’s time to make love, she doesn’t want to.* (Female CHW, 42 years old)

Side effects affected marital relationships and women’s ability to work.

Women managed side effects through a variety of techniques. Some women expressed the importance of “perseverance” in the face of challenges:

*There are some people who have side effects; they no longer have periods, some gain a lot of weight, they have problems in their bodies, but they persevere.* (Female community member, 37 years old)

Others spoke with their CHW or returned to the health center to switch methods:

*Community health workers educate people about family planning and people choose the method they want to use. If they find that the method they first chose doesn’t work well for them, community health workers let them choose another method until they find the method that works best with their body.* (Male community member, 45 years old)

Yet many women felt the side effects were too great and eventually discontinued family planning use, sometimes after trying several different methods:

*[Some] women use injections. Then they change and start using pills. Another woman now has an implant, but she also wants to discontinue. She wants to discontinue because she is bleeding excessively. When she has her period it never seems to end.* (Female CHW, 48 years old)

Participants’ experiences of side effects were often shaped by rumors and misinformation in the community, coupled with limited opportunities to receive in-depth information and counseling from providers. The combination of negative experiences, misinformation, and rumors contributed to fears that even popular methods of family planning such as injectables could have harmful long-term consequences, such as infertility, cancer, or death:

*What I can tell you is that there were some rumors among women. Women were saying that family planning causes cancer and that there was a program to inject women with cervical cancer … those rumors spread and many women did not want to try family planning.* (Female CHW, 42 years old)

Rumors and misinformation contributed to fears of contraceptive methods.

Upon hearing about negative side effects and potential life-threatening illnesses linked to family planning, many women were wary of joining the family planning program. As 1 CHW explained:

*There are people who fear using family planning because of information they get from their friends about side effects. When 1 woman experiences a side effect, she may go around to her neighbors and friends talking to them about her problem, and this makes them fear joining family planning.* (Male CHW, 34)

## DISCUSSION

In interviews, participants shared both the benefits and challenges of family planning in the context of desires for large families, social pressures, gender roles, side effects, and barriers to accessing services. Our study reinforces that individual and community perceptions exert a strong influence on reproductive health and family planning choices,^19^^–^^21^ and that understanding providers’ and communities’ perspectives is critical if family planning program priorities and messaging are to respond to the needs of the local context.^22^

Participants spoke openly about the numerous benefits of family planning to promote poverty eradication, familial development, and better health. The fact that these positive statements of family planning often echoed the messages shared by local leaders, in national campaigns, and on radio broadcasts does suggest that public health information campaigns on family planning are raising awareness in this rural district of Rwanda. However, awareness is not sufficient to ensure behavior change, as is evident in participants’ reflections on the pragmatic and sociocultural challenges of adopting and adhering to family planning. This finding is not uncommon among family planning programs.^23^

Participants shared desires for larger families as a sign of prestige or protection against unforeseen disaster and child mortality. This echoes literature that has shown a strong replacement effect on fertility in Rwanda following the 1994 genocide.^24^ Further, although Rwanda has achieved impressive declines in child mortality, in 2010 16.6% of women had experienced 2 or more child deaths.^7^

Over the past decade, Rwanda’s family planning program has expanded significantly in national health centers^17^ and through national policies that require family planning services to be offered free of charge in order to eliminate financial barriers. CHWs have taken on additional responsibilities to educate communities about family planning, and in pilot districts, CHWs are able to administer some contraceptive methods. Yet structural factors such as hidden costs associated with services, transportation fees, and supply shortages created barriers to sustained enrollment in the family planning program for participants in this study. These barriers are noted throughout sub-Saharan Africa in areas where human resources for health are below recommended thresholds and health system infrastructure remains weak.^25^ And while Rwanda has initiated a model program for the scale-up of human resources for health,^26^ the country still requires additional health workers to meet national health worker needs. In our study, these human resource shortages were reflected in the long wait times and short appointments with service providers, which often led to insufficient counseling on family planning.

While Rwanda’s family planning program has made great strides to improve access to family planning, structural barriers still persist.

In addition, the reach and quality of services can be strengthened by providing training and mentorship for CHWs and health care providers alike to facilitate the delivery of appropriate counseling and services, so that women can make informed choices when selecting contraceptive methods and better anticipate side effects. CHWs specifically could be provided with side effect management protocols and supplies to distribute throughout their communities to help women cope (e.g., sanitary napkins and pain relievers).

Training health workers to provide appropriate counseling, including proper management of side effects, is critical.

Factors contributing to stock-outs of reproductive commodities have recently been analyzed in Rwanda,^27^ and efforts to address these areas will be needed to narrow gaps between national policy and local implementation. Additionally, assuring family planning services are truly free of charge to clients will be instrumental to improving coverage. Clarifying and enforcing national policies regarding payment at the local level are essential to ensure that unnecessary costs are not incurred by program recipients and to encourage individuals to seek care at health centers.

Given these structural challenges, when women faced persistent and significant side effects, many chose to discontinue use, a common theme in the literature on family planning in low-resource settings.^28^^,^^29^ Negative experiences were often shared through social networks and affected the willingness of others in the community to try contraceptive methods, exacerbating fears of potential long-term negative health impacts. Such fears have been documented as a common reason for contraceptive discontinuation in similar settings.^30^^,^^31^

Interpersonal relationships, particularly with male partners, exerted significant influence on reproductive health decision making, with male partners often exercising control over women’s reproductive choices, a finding echoed by researchers implementing similar programs in diverse countries throughout sub-Saharan Africa.^32^^,^^33^ Yet while male partners exerted control over reproductive choices, family planning was often seen as a matter that concerned only women. Recognizing the critical importance and significant benefits of engaging men and boys in reproductive health and family planning efforts,^34^ the Government of Rwanda has initiated successful programs to involve male partners in reproductive health services, which could be considered for expansion. These include a national program for the prevention of mother-to-child transmission of HIV, in which 81% of men in 2010 took advantage of HIV counseling and testing services during their female partner’s antenatal care visits.^35^

Expanding efforts to engage men and boys in reproductive health programs could be considered.

Further, closing the gap between national policies that target all women of reproductive age (15–49) and local realities that limit the access of young and unmarried women, is essential to ensuring that young women, in and out of school, have access to information and services and the ability to make informed choices about their SRH. Youth-friendly health centers and targeted national messaging can also make an important contribution toward improving access to SRH information and services for young people.

### Limitations

These results should be interpreted with limitations in mind. While this study offers a broad overview of perspectives on reproductive health and family planning for a large sample of participants in southern Kayonza, Rwanda, our findings should not be generalized beyond the study population. Although we have described common perspectives shared by many participants in our study, these participants varied significantly in key social and demographic factors such as age and occupation, in their personal experiences and beliefs, and accordingly, in the responses they provided during interviews. Unfortunately these nuances cannot be adequately explored in a single journal article, and further sub-analysis, for example, stratified by age and gender, is outside the scope of this paper. We did, however, identify in this first phase of research a critical need to explore the perspectives of young people in-depth, which was addressed through a second round of data collection with adolescents, the results of which are forthcoming. Finally, the position of the research team must be acknowledged. Data collectors were hired independently of the service delivery personnel of PIH-IMB, and participants were assured throughout the interview and during the informed consent process that their responses would be held in confidence and would not affect the services provided; however, it was impossible to entirely divorce ourselves from any association with this public-private partnership, and such association likely affected the information that was disclosed.

## CONCLUSIONS

Although Rwanda has achieved impressive gains in contraceptive coverage, and people generally are aware of the benefits of family planning, our findings suggest that awareness is not always enough to change behavior and expressed desires regarding fertility and family size. Rwanda has demonstrated strong political will to promote family planning as a pillar of its approach to sustainable development,^36^ and the country recently adopted a national target to increase coverage of voluntary contraceptive use among women of reproductive age to 72% by 2018.^37^ As Rwanda continues to refine its policies to achieve these goals, it will be critical to ensure that national messaging campaigns address local perceptions and engage men and boys, and that strategies are adopted to mitigate the very real social, economic, and health challenges that women, including young and unmarried women, face in accessing and continuing to use family planning. Without such strategies, it is likely that family planning services will fail to reach all those who could benefit from these services.

## Notes

**Peer Reviewed**
